# A Formaldehyde-Free, Soy Protein-Based Adhesive with P-N-Si Synergistic Flame Retardancy for Plywood Application

**DOI:** 10.3390/polym18192312

**Published:** 2026-09-22

**Authors:** Han Yang, Nicolas Gascoin, Liping Jiang, Xiaofeng Zhu, Xiang Huang, Khaled Chetehouna, Ruihua Guo

**Affiliations:** 1 School of Materials Science and Engineering, Zhejiang University, Hangzhou 310027, China; 2 Hangzhou Municipal Fire and Rescue Bureau, Hangzhou 310008, China; jiangsir119119@163.com (L.J.); huangxiang119119@163.com (X.H.); 3 PRISME UR 4229, F-18000 Bourges, France; nicolas.gascoin@insa-cvl.fr (N.G.); khaled.chetehouna@insa-cvl.fr (K.C.); 4 Institute for Smart Ageing, Beijing Academy of Science and Technology, Beijing 100089, China; zxf_402@163.com; 5 Beijing Key Laboratory of AgeTech and Health Engineering, Beijing Academy of Science and Technology, Beijing 100089, China

**Keywords:** soy protein adhesive, P-N-Si synergism, intumescent flame retardant, plywood

## Abstract

To overcome the flammability and formaldehyde-emission problems associated with plywood, a novel soy protein-based intumescent flame-retardant adhesive with phosphorus–nitrogen–silicon (P-N-Si) synergism (C@Si@APP) was developed in this study. C@Si@APP was initially prepared by coating silane-modified ammonium polyphosphate with collagen. Subsequently, C@Si@APP was combined with lignin and adenine and incorporated into a soy protein, collagen, and isocyanate (MDI)-based adhesive matrix to fabricate a flame-retardant adhesive for eucalyptus plywood. Cone calorimeter (CONE) analysis demonstrated that the flame-retardant plywood bonded with SPI/C/MDI/C@SiAPP18 achieved the most effective flame-retardant and smoke-suppression performance, and the peak heat release rate, total heat release, and total smoke production were reduced by 23.5%, 20.7%, and 43.9%, respectively, and the release rates of CO and CO_2_ were also effectively suppressed. Mechanistic analysis indicated that the enhanced flame retardancy was mainly governed by a condensed-phase mechanism, in which phosphorylation and siloxane crosslinking promoted the formation of a compact and thermally stable char layer that protected the underlying material, whereas a gas-phase contribution involving the dilution of flammable volatiles by non-combustible gases (NH_3_, H_2_O, and CO_2_) and possible radical scavenging by phosphorus-containing species is proposed as a plausible complementary pathway inferred from the TGA-FTIR results and the previous literature.

## 1. Introduction

Plywood is primarily manufactured by bonding wood veneers in alternating layers with adhesives, and its symmetrical structure together with its stable performance has enabled widespread application in building decoration, packaging and transportation, as well as vehicle and ship manufacturing. However, plywood is highly combustible by nature, and its burning process generates considerable heat and dense smoke, thereby posing serious threats to property and human safety. Existing flame-retardant technologies for plywood can generally be classified into two approaches: veneer impregnation modification and adhesive modification. Veneer impregnation commonly employs atmospheric or pressure-assisted treatments to introduce water-soluble flame-retardant solutions into the wood structure. The formulations used have gradually evolved from single-component flame retardants, such as ammonium polyphosphate and ammonium dihydrogen phosphate, to multi-component synergistic systems [1,2]. For example, the combination of bio-based furfuryl alcohol (FA) with phosphorus- and boron-containing flame-retardant formulations, including ammonium phosphate and boric acid, has been reported to increase the limiting oxygen index (LOI) [1,3]. Furthermore, cyclic impregnation processes employing alternating melamine (MEL) and amino trimethylene phosphonic acid (ATMP) have effectively alleviated the leaching problems associated with conventional flame retardants [4,5]. Despite the significant enhancement in flame resistance achieved by veneer impregnation, these techniques generally involve complex processing procedures, high chemical consumption, and lengthy production and curing times. By contrast, adhesives are essential components of all panels and are applied immediately before hot pressing; therefore, modification of the adhesive itself can avoid these additional processing steps and can be directly integrated into existing manufacturing lines. Accordingly, direct flame-retardant modification of adhesives has become a more practical and industrially compatible strategy.

Conventional flame-retardant plywood adhesives have mainly been developed from urea–formaldehyde (UF), melamine–urea–formaldehyde (MUF), and phenol–formaldehyde (PF) resins [6,7,8,9]. Nevertheless, these adhesives inevitably release toxic formaldehyde during service. Accordingly, the development of formaldehyde-free flame-retardant adhesives has become an important direction for the wood-based panel industry. Currently, the exploration of formaldehyde-free adhesive systems has mainly focused on utilizing inorganic adhesives (e.g., silicon- and magnesium-based systems) to provide thermal barriers [10], and on developing bio-based adhesives [11,12,13,14,15]. However, inorganic adhesive systems are generally brittle, difficult to process, and have lower bonding strength than organic resins, which restricts their wider application. Among the various bio-based resources, soy protein has emerged as a research focus for formaldehyde-free adhesives by virtue of its wide availability, low cost, and abundant reactive functional groups. However, pure soy protein exhibits inherent defects, including low crosslinking density, poor water resistance, and high flammability, which severely restrict its application in the wood-based panel industry. Therefore, efficient flame-retardant modification is urgently required to overcome this bottleneck.

In recent years, flame-retardant modification of soy protein adhesives has mainly relied on synergistic strategies involving phosphorus-, nitrogen-, and silicon-containing functional components [16,17,18,19].

Ammonium polyphosphate (APP) is an effective phosphorus- and nitrogen-rich flame retardant; however, its poor compatibility with polymer matrices often deteriorates the overall material performance, making surface modification necessary. Common approaches include surface treatment with tetrakis(hydroxymethyl)silane (THMS), as used for the Si@APP precursor in this study [20], and microencapsulation with biomacromolecules, as applied here through collagen coating [21,22]. Previous studies have demonstrated that polysiloxane-modified APP can significantly reduce the total heat release and total smoke production of low-density polyethylene while causing only a 0.76% reduction in tensile strength [23]. However, because this evidence was obtained from a non-polar polyolefin matrix, its applicability to the polar, protein-rich soy adhesive used in the present study cannot be assumed and was therefore verified directly by the FTIR/XPS characterization of C@Si@APP in Section 3.1.1. These findings indicate that surface chemical modification or physical microencapsulation can effectively enhance the interfacial compatibility of APP, thereby providing a practical modification strategy for its application in soy protein adhesives. Furthermore, to improve the flame-retardant efficiency of APP while satisfying the demand for sustainable materials, the combination of APP with bio-based compounds to construct intumescent flame-retardant (IFR) systems has emerged as an active area of research [24].

In this work, a bio-based composite adhesive system consisting of soy protein, collagen, and isocyanate (MDI) was successfully established. While maintaining its formaldehyde-free and partially bio-based characteristics, collagen-coated silane-modified ammonium polyphosphate (C@Si@APP), together with lignin and adenine, was incorporated to construct a P-N-Si synergistic intumescent flame-retardant system. In this system, C@Si@APP, adenine, and lignin act as the acid, gas, and carbon sources, respectively, and are introduced directly into the adhesive rather than into the wood veneer. This dual silane/collagen modification of APP, combined with in-adhesive P-N-Si synergism, distinguishes this work from previous single-modification or veneer-impregnation strategies. The phosphorus and silicon components were expected to promote char formation and condense into a compact Si-O-Si/Si-C protective layer, the nitrogen-rich adenine was expected to strengthen the char through a P-N network while releasing non-combustible diluent gases [25], and lignin was expected to function as an additional carbon source [26,27,28], together achieving flame retardancy through combined condensed- and gas-phase mechanisms. This strategy effectively addressed the excessive formaldehyde emissions associated with conventional plywood adhesives, as well as the limited thermal stability and flame-retardant performance of soy protein adhesives. The prepared flame-retardant adhesive satisfied the bonding-strength requirements for Class II plywood and exhibited low heat release rates and reduced total smoke production during cone calorimeter testing. The novelty of this work can be summarized in three aspects: (i) the dual silane/collagen surface modification of APP, which simultaneously improves the interfacial compatibility of APP and introduces additional char-forming and gas-source components; (ii) the integration of the P–N–Si synergistic intumescent system entirely within a formaldehyde-free soy protein adhesive layer, which avoids veneer impregnation or any additional surface pretreatment; and (iii) the direct demonstration of flame-retardant, smoke-suppression, and Class II bonding performance at the plywood level under realistic cone calorimeter conditions.

In comparison with the most recent studies, the system proposed in this work offers a distinct balance between bonding performance and fire safety. For instance, Zhang et al. [29] reported a soy protein adhesive crosslinked with a POSS–urushiol core–shell hybrid and sodium borate, which reduced the PHRR of the cured adhesive by 25.4% and provided dry and wet shear strengths of 2.46 and 0.74 MPa, respectively; however, the flame retardancy was evaluated on the adhesive itself, and the fire behavior of the bonded plywood was not assessed. Yang et al. [15] prepared flame-retardant plywood by combining a dialdehyde cellulose-based adhesive with an additional phytic acid/SiO_2_ veneer coating, achieving a dry bonding strength of 1.6 MPa and a limiting oxygen index of 38.5%; this high performance nevertheless relied on an extra surface-treatment step applied to the veneers. In contrast, the P–N–Si intumescent system in this work is introduced entirely within the adhesive layer, requiring no veneer pretreatment, while the resulting plywood simultaneously satisfied the Class II bonding-strength requirement (dry and wet shear strengths of 1.15 and 0.81 MPa, respectively) and exhibited reductions of 23.5%, 20.7%, and 43.9% in PHRR, THR, and TSP, respectively, together with suppressed CO and CO_2_ release during cone calorimeter testing. This study provides a promising strategy for the development of flame-retardant, formaldehyde-free soy protein adhesives for plywood manufacturing.

## 2. Materials and Methods

### 2.1. Materials

Diphenylmethane diisocyanate (MDI, industrial grade, -NCO content 31% ± 0.5%, viscosity ~200 mPa·s at 25 °C) was obtained from Wanhua Chemical Group Co., Ltd, Yantai, China. Soy protein powder (industrial grade, protein content ≥90%, moisture content ≤8 wt%) was purchased from Beijing Kaitai New Century Biotechnology Co., Ltd, Beijing, China. Collagen powder (protein content ≥85%, ash content ≤3 wt%) was supplied by Hebei Huachen Biotechnology Co., Ltd, Cangzhou, China. Ammonium polyphosphate (APP, type II, analytical grade, degree of polymerization n > 1000, P_2_O_5_ content ≥71 wt%, nitrogen content ≥14 wt%) and tetrakis(hydroxymethyl)silane-modified ammonium polyphosphate (Si@APP, industrial grade, surface-treated with tetrakis(hydroxymethyl)silane, THMS) were purchased from Shandong Changsheng Flame Retardant New Material Co., Ltd, Dezhou, China. Melamine (MEL, analytical grade, purity ≥99%) and pentaerythritol (PER, analytical grade, purity ≥98%) were obtained from Sinopharm Chemical Reagent Co., Ltd, Shanghai, China. Adenine (analytical grade, purity ≥99%) and alkali-extracted lignin (industrial grade, ash content 10–25%) were purchased from Aladdin Reagent (Shanghai) Co., Ltd, Shanghai, China. Eucalyptus veneers (density: 0.62 g/cm^3^, thickness: 1.7 mm, moisture content: 20%) and decorative veneers (density: 0.62 g/cm^3^, thickness: 0.4 mm, moisture content: 22%) were supplied by Jiangsu Guanyuan Home Furnishing Co., Ltd, Suqian, China.

### 2.2. Synthesis of Flame Retardant (C@Si@APP)

First, 10 g of Si@APP was dispersed in 100 mL of boiling water to obtain a suspension. A collagen solution was then prepared by dissolving 20 g of collagen in 80 mL of deionized water. Subsequently, 100 mL of the 20 wt% collagen solution was slowly introduced into the Si@APP suspension and stirred using a JJ-1 electric stirrer (Changzhou Guohua Electric Appliance Co., Ltd., Changzhou, China) for 3 min to promote sufficient adsorption. The coated product, C@Si@APP, was then collected by centrifugation using a TG16-WS high-speed centrifuge (Xiangyi Centrifuge Instrument Co., Ltd., Changsha, China) at 3500 rpm for 10 min. The resulting pale-yellow viscous product was dried in a DHG-9070A electric blast drying oven (Shanghai Yiheng Scientific Instrument Co., Ltd., Shanghai, China) at 70 °C for 12 h and subsequently pulverized using an FW100 high-speed universal crusher (Taisite Instrument Co., Ltd., Tianjin, China) to obtain C@Si@APP powder with a particle size of approximately 15–25 μm, as shown in Figure 1.

### 2.3. Preparation of the Soy Protein-Based Composite Adhesive, Flame-Retardant Adhesive, and Plywood

#### 2.3.1. Soy Protein-Based Composite Adhesive

The composite protein component was first pre-mixed from soy protein and collagen powders at a mass ratio of 8:2 (the optimization of this ratio is discussed in Section 3.2). The pre-mixed protein powder was added to deionized water in a reaction kettle and mechanically stirred at 50 °C for 1 h using a JJ-1 electric stirrer (Changzhou Guohua Electric Appliance Co., Ltd., Changzhou, China); a NaOH solution was then added dropwise to adjust the pH to 10.0, followed by further mechanical stirring for 1 h to obtain a protein dispersion with a solid content of 30 wt%. The dispersion was subsequently placed in an ice-water bath (10–15 °C), and MDI (10 wt%) was added slowly in two batches at a 5 min interval under low-speed stirring to achieve preliminary dispersion. After the addition was completed, the stirring speed was increased to 800 rpm and maintained at room temperature for about 15 min until a fine, lump-free, and uniformly dispersed homogeneous viscous liquid was formed, followed by vacuum defoaming, thereby yielding the composite soy protein adhesive. The resulting composite soy protein adhesive is hereafter denoted as SPI/C/MDI.

#### 2.3.2. Flame-Retardant Adhesive and Plywood

The composite soy protein adhesive was mixed with the flame retardant at a ratio of 7:3 and stirred with a high-speed mixer at 800 r/min for about 15 min to ensure adequate dispersion and uniformity. Four flame-retardant composite soy protein-based adhesives were prepared according to the different formulations listed in Table 1.

The four adhesives prepared above, namely SPI/C/MDI, SPI/C/MDI/C@SiAPP18, SPI/C/MDI/C@SiAPP15, and SPI/C/MDI/IFR, were uniformly applied to both surfaces of each eucalyptus core veneer using a single-side spreading process at a glue application rate of 180 g/m^2^. Five core eucalyptus veneers (400 mm × 400 mm × 1.7 mm) and two decorative veneers (400 mm × 400 mm × 0.4 mm) were assembled with the grain directions of adjacent layers perpendicular to one another, carefully aligned and arranged. The assembly was cold-pressed for 30 min, followed by hot-pressing for 15 min at 120 °C and 1.0–1.5 MPa to fully cure the adhesive and form a stable bonding structure. After hot pressing, the panels were naturally cooled at room temperature for 8 h. Prior to testing, the finished plywood panels with a thickness of approximately 10 mm were conditioned at 25 °C and 60% relative humidity for 24 h before use.

### 2.4. Characterization

#### 2.4.1. Thermogravimetric Analysis (TGA)

The thermal stability of the flame retardants and flame-retardant adhesives was evaluated using a NETZSCH STA 2500 thermogravimetric analyzer, by NETZSCH-Gerätebau GmbH, located in Selb, Bavaria, Germany. The sample mass was about 8–10 mg. Each sample was first held at 100 °C for 5 min and then heated to 800 °C at a rate of 10 °C/min under a nitrogen atmosphere.

#### 2.4.2. Cone Calorimeter Test

The combustion behavior of the plywood was assessed using a cone calorimeter (FTT Cone2+, Fire Testing Technology, East Grinstead, UK) under an external heat flux of 50 kW/m^2^, according to ISO 5660-1 [30]. Specimens with dimensions of 100 mm × 100 mm were cut from the finished seven-layer plywood panels (approximately 10 mm in thickness). The seven-layer plywood was selected to better simulate the fire response of real commercial plywood products, as it more closely represents actual plywood in terms of thickness and heat-transfer behavior. Prior to testing, all specimens were conditioned at 25 °C and 60% relative humidity for 24 h. Each specimen was wrapped in aluminum foil on the back and edges, leaving only the top surface exposed, and was placed horizontally in the specimen holder with the exposed surface facing the conical heater. Three replicate specimens were tested for each formulation. The reported data are the mean values of the three replicate specimens, and the data-processing and statistical analysis procedures are described in Section 2.5.

#### 2.4.3. Shear Strength Test

The dry and wet shear strengths of the plywood were evaluated according to the Chinese national standard GB/T 17657-2013 [31], which technically aligns with the international standard ISO 12466-1 [32]. Specimens (length × width: 100 mm × 25 mm; bonding area: 25 mm × 25 mm) were cut from the finished plywood panels. The tests were performed at room temperature using a universal testing machine (LD25.504, LISHI, Shanghai, China) at a crosshead speed of 10 mm/min, and six replicate specimens were tested for each formulation. For the wet shear strength measurement, the specimens were immersed in deionized water at 63 °C for 3 h and then cooled naturally to 25 °C prior to testing. The measured values were evaluated against the requirement specified in GB/T 9846-2015 [33].

#### 2.4.4. TGA-FTIR

A NETZSCH STA 2500 thermogravimetric analyzer coupled with a Thermo-Nicolet iS50 spectrometer (Thermo Fisher Scientific, Middleton, WI, USA) was used to determine the chemical structure and composition of the thermo-oxidative decomposition products released from the adhesives (SPI/C/MDI and SPI/C/MDI/C@SiAPP18). The adhesive samples were heated from 25 °C to 800 °C at a rate of 10 °C/min in air, in order to analyze the oxidative degradation behavior of the adhesives during combustion, with spectra recorded over the range of 600–4000 cm^−1^ at a resolution of 4 cm^−1^.

#### 2.4.5. Fourier-Transform Infrared Spectroscopy (FTIR)

Spectra were acquired using a Thermo-Nicolet iS50 FTIR spectrometer over the range of 4000–400 cm^−1^ at a resolution of 4.0 cm^−1^.

#### 2.4.6. Microstructural Characterization (SEM-EDS)

The morphology of the char residues was observed using a ZEISS Gemini SEM360 scanning electron microscope (Carl Zeiss Microscopy, Oberkochen, Germany) equipped with an energy-dispersive spectrometer, which was also used to examine the elemental distribution on the char surface.

#### 2.4.7. X-Ray Photoelectron Spectroscopy (XPS)

The chemical composition of the char residues after cone calorimeter testing was characterized using a Thermo Scientific ESCALAB 250Xi X-ray photoelectron spectrometer (Thermo Fisher Scientific, East Grinstead, UK). The binding-energy scale was calibrated with reference to the C1s peak of adventitious carbon at 284.8 eV, and the high-resolution spectra were fitted with Gaussian–Lorentzian functions after Shirley background subtraction using the Avantage software (version 6.6.0).

#### 2.4.8. Raman Spectroscopy

Raman spectra were collected using a Jobin-Yvon Labor Raman HR-800 spectrometer (HORIBA Jobin Yvon, Longjumeau, France) equipped with a 514.5 nm Ar+ laser at a power of 5–50 mW.

### 2.5. Statistical Analysis

All data analyses were performed using Microsoft Excel 2019. Experimental data are uniformly expressed as the mean ± standard deviation (SD). One-way analysis of variance (ANOVA) coupled with Tukey’s post hoc test was applied for statistical comparison among groups. Differences were regarded as significant when *p* < 0.05.

## 3. Results and Discussion

### 3.1. Characterization of the Flame Retardant and Flame-Retardant Adhesive

#### 3.1.1. Chemical Characterization of the Flame Retardant

To verify the successful synthesis of C@Si@APP, the FTIR spectra of APP, Si@APP, collagen, and C@Si@APP were compared, as presented in Figure 2. The O-H/N-H stretching band at 3285 cm^−1^ observed in C@Si@APP was also detected in both Si@APP and collagen, indicating that hydroxyl and amino groups derived from the two precursors were retained in C@Si@APP. The characteristic collagen bands at 1651 cm^−1^ (C=O, amide I) and 1541 cm^−1^ (C-N, amide II) were clearly observed in C@Si@APP without an obvious shift, indicating that collagen was associated with the surface of Si@APP. In addition, the bands at 1251 and 1080 cm^−1^, assigned to P=O stretching vibrations, and the band at 880 cm^−1^, attributed to P-O-P skeletal vibration, were highly consistent between Si@APP and C@Si@APP, indicating that the phosphate backbone was preserved after silane modification and collagen coating. Thus, C@Si@APP retained the phosphate-related structural features of Si@APP (P=O, P-O-P) and the protein-related functional groups of collagen (C=O, C-N), confirming effective interfacial association between Si@APP and collagen during compounding while maintaining the principal functional groups.

The elemental composition of C@Si@APP and Si@APP was further examined by XPS. As shown in Figure 2, the surface carbon content increased by approximately two-fold after collagen was coated onto Si@APP, rising from 28.4% for Si@APP to 57.6% for C@Si@APP, based on the survey spectra shown in Figure 2b. Despite the formation of the surface coating, P, N, and Si were still retained in the system. In the C 1s spectrum, the peaks at 287.9, 286.1, and 284.8 eV were assigned to C–N, C–O/C=O, and C–C, respectively. In the N 1s spectrum, the peaks at 399.8, 401.3, and 402.0 eV were assigned to C–N, N–C=O, and NH_4_^+^, respectively. The C–N and N–C=O signals further supported the introduction of collagen. The P–O (133.7 eV) and P=O (134.3 eV) peaks in the P 2p spectrum, together with the Si–C (102.0 eV) and Si–O (102.8 eV) peaks in the Si 2p spectrum, indicated that the phosphate and silicon-containing structures were retained after collagen coating. These FTIR and XPS results are consistent with the formation of a collagen coating on the surface of Si@APP and confirm the successful preparation of C@Si@APP.

#### 3.1.2. Thermogravimetric Analysis of the Flame Retardants and Flame-Retardant Adhesives

As shown in Figure 3 and Table 2, the TG-DTG curves of the four flame retardants displayed multistage thermal decomposition behavior. The temperatures at 5% weight loss (T_5%_) of APP and Si@APP were 329 °C and 325 °C, respectively, whereas collagen and C@Si@APP exhibited lower T_5%_ values of 175 °C and 188 °C, respectively. The lower T_5%_ of C@Si@APP was mainly attributed to the collagen component, which showed an early first decomposition peak (T_max1_) at 80 °C, likely corresponding to the loss of bound water and the initial denaturation of the collagen coating. This early-stage mass loss did not appear to compromise the flame-retardant process and may instead have contributed to the initiation of subsequent char formation. Regarding char yield, C@Si@APP retained 39.2% of its mass at 800 °C (W_exp-800 °C_), which was about 34.7%, 58.7%, and 84.9% higher than those of APP, collagen, and Si@APP, respectively, demonstrating its superior char-forming capacity. According to the DTG curves, APP and Si@APP exhibited first decomposition peaks (T_max1_) at 322 °C and 328 °C, with corresponding peak mass loss rates (PMLR) of 2.03%/°C and 1.69%/°C, and second decomposition peaks (T_max2_) at 613 °C and 757 °C, with PMLR values of 5.84%/°C and 5.92%/°C, respectively. Collagen showed decomposition peaks at 80 °C and 315 °C, with PMLR values of 0.76%/°C and 5.33%/°C. C@Si@APP displayed decomposition peaks at 280 °C and 542 °C, with PMLR values of 3.63%/°C and 0.97%/°C. Although the T_5%_ of C@Si@APP was lower than those of APP and Si@APP, its decomposition rate in the high-temperature region was markedly reduced, indicating that the integration of Si@APP with collagen enhanced the char-forming ability of C@Si@APP at elevated temperatures.

The thermal stability of the flame-retardant adhesives is shown in Figure 3 and Table 2. The T_5%_ values of SPI/C/MDI/C@SiAPP18 and SPI/C/MDI were 238 °C and 236 °C, respectively, showing no substantial difference, which indicates that the flame-retardant treatment had only a limited influence on early-stage thermal stability. In terms of char yield, the W_exp-800 °C_ of SPI/C/MDI/C@SiAPP18 was 44.2%, about 53.5% higher than that of SPI/C/MDI, indicating enhanced carbonization ability. According to the DTG curves, SPI/C/MDI showed a first decomposition peak (T_max1_) at 92 °C with a PMLR of 0.36%/°C, whereas the T_max1_ of SPI/C/MDI/C@SiAPP18 shifted to 88 °C with a lower PMLR of 0.22%/°C. The reduced early-stage PMLR suggests that the flame-retardant components suppressed initial thermal decomposition, possibly by promoting dehydration, crosslinking, or preliminary char formation during heating. The T_max2_ of SPI/C/MDI appeared at 326 °C with a PMLR of 4.1%/°C, whereas the T_max2_ of SPI/C/MDI/C@SiAPP18 was delayed to 335 °C with a PMLR of 4.2%/°C, indicating that the flame-retardant treatment postponed the main decomposition stage and promoted the development of a protective char layer.

### 3.2. Bonding Performance of the Plywood

To determine the optimal soy protein-to-collagen ratio, composite soy protein adhesives with soy protein:collagen mass ratios of 9:1, 8:2, and 7:3 (denoted as SPI/8C/MDI, SPI/16C/MDI, and SPI/24C/MDI, respectively) were prepared, with a pure soy protein adhesive (SPI) as the control, and the dry and wet shear strengths of the corresponding plywood were measured. The dry and wet shear strengths of the SPI-bonded plywood were 1.18 MPa and 0.49 MPa, respectively. As the collagen content increased, the bonding strength first increased and then decreased: the dry and wet shear strengths were 1.27 MPa and 0.91 MPa for SPI/8C/MDI, 1.36 MPa and 0.97 MPa for SPI/16C/MDI, and 1.30 MPa and 0.92 MPa for SPI/24C/MDI. This trend can be attributed to the covalent crosslinking network formed between the -NCO groups of MDI and the amino and hydroxyl groups of the protein chains, together with the physical reinforcement provided by the fibrous collagen structure, whereas an excessive collagen content reduced the effective crosslinking density. All three composite adhesives exceeded the minimum wet shear strength of 0.7 MPa required for Class II interior-use plywood in GB/T 9846-2015, and the formulation with a soy protein:collagen ratio of 8:2 (SPI/16C/MDI) exhibited the highest dry and wet shear strengths; it was therefore selected as the base adhesive for the subsequent flame-retardant modification.

The influence of flame-retardant incorporation on the bonding performance was further evaluated for the plywood bonded with SPI, the composite soy protein adhesive (adhesive code: SPI/C/MDI), the adhesive containing 30 parts of C@Si@APP alone (denoted as SPI/C/MDI/C@SiAPP30), and SPI/C/MDI/C@SiAPP18. The plywood bonded with SPI showed a dry shear strength of 1.18 MPa and a wet shear strength of only 0.49 MPa, because the SPI molecules rely mainly on weak hydrogen bonding, resulting in poor cohesive strength and water resistance. After the introduction of collagen and MDI, the dry and wet shear strengths of the plywood bonded with the composite adhesive increased to 1.36 MPa and 0.97 MPa, respectively, and wood failure predominated in the fractured specimens, indicating that the bonding strength exceeded the strength of the wood itself. When 30 parts of C@Si@APP were incorporated alone, the dry and wet shear strengths decreased to 1.07 MPa and 0.70 MPa, respectively; this decrease is mainly attributed to the high loading of rigid inorganic particles, which diluted the effective bonding component per unit volume, disrupted the continuity of the adhesive layer, and formed weak interfacial regions through which water could more easily penetrate under wet conditions. With the combined incorporation of 18 parts of C@Si@APP, 6 parts of adenine, and 6 parts of lignin (SPI/C/MDI/C@SiAPP18), the dry and wet shear strengths recovered to 1.15 MPa and 0.81 MPa, respectively, because the amino groups of adenine could form hydrogen bonds with the matrix or participate in crosslinking with residual -NCO groups, while the phenolic hydroxyl groups of lignin constructed a dense hydrogen-bonding network and provided physical reinforcement, together compensating for the strength loss caused by the flame retardant. Although the wet shear strength of this formulation was slightly lower than that of the composite adhesive matrix, it still satisfies the wet-strength requirement (≥0.7 MPa) for Class II interior-use plywood specified in GB/T 9846-2015, demonstrating its potential for practical applications.

### 3.3. Combustion Behavior of the Plywood

Cone calorimetry can simulate the combustion behavior of materials under realistic fire conditions and provides a comprehensive basis for evaluating flame-retardant and smoke-suppression performance. Figure 4 shows the heat release rate (HRR), total heat release (THR), smoke production rate (SPR), and total smoke production (TSP) curves of the different flame-retardant plywoods, and the corresponding data are summarized in Table 3. The HRR curve of SPI/C/MDI displayed a typical two-peak profile, with a first peak heat release rate (PHRR_1_) of 291 kW/m^2^, a second peak heat release rate (PHRR_2_) of 214 kW/m^2^, and a total heat release (THR) of 63.59 MJ/m^2^. The HRR and THR values of SPI/C/MDI/C@SiAPP18, SPI/C/MDI/C@SiAPP15, and SPI/C/MDI/IFR were all substantially decreased. SPI/C/MDI/C@SiAPP18 exhibited a PHRR_1_ of 222.6 kW/m^2^ and a PHRR_2_ of 116 kW/m^2^, with a THR of 50.45 MJ/m^2^. SPI/C/MDI/C@SiAPP15 showed a PHRR_2_ of 203 kW/m^2^ and a THR of 55.61 MJ/m^2^, both of which were lower than those of SPI/C/MDI. SPI/C/MDI/IFR presented a PHRR_2_ of 191 kW/m^2^ and a THR of 53.58 MJ/m^2^, indicating that SPI/C/MDI/C@SiAPP18 performed better than the conventional flame-retardant system in reducing combustion intensity and cumulative heat release. In addition, all three flame-retardant systems reached PHRR_1_ at about 40–50 s, which was close to the value observed for SPI/C/MDI. However, the time required to reach PHRR_2_ differed markedly: SPI/C/MDI reached the second heat release peak at 650 s, noticeably earlier than the three flame-retardant plywoods. SPI/C/MDI/C@SiAPP18 showed the best performance in this respect, delaying the second peak by 360 s compared with SPI/C/MDI. Therefore, all three flame-retardant systems effectively suppressed heat release during plywood combustion, whereas SPI/C/MDI/C@SiAPP18 produced the greatest reductions in PHRR and THR, especially by inhibiting the second heat release peak in the middle-to-late combustion stage. Although SPI/C/MDI/IFR showed a slightly stronger effect than SPI/C/MDI/C@SiAPP15 in reducing PHRR_2_, its total heat release was still higher than that of SPI/C/MDI/C@SiAPP18.

With respect to smoke release, SPI/C/MDI showed a total smoke production (TSP) of 3.42 m^2^, with a first peak smoke production rate (PSPR_1_) of 0.041 m^2^/s and a second peak smoke production rate (PSPR_2_) of 0.044 m^2^/s, both of which were higher than those of the three flame-retardant plywoods. SPI/C/MDI/C@SiAPP18 exhibited a TSP of 1.92 m^2^, which was 43.9% lower than that of SPI/C/MDI, while PSPR_1_ and PSPR_2_ decreased by 14.6% and 79.5%, respectively. SPI/C/MDI/C@SiAPP15 showed a TSP of 2.37 m^2^, 30.7% lower than that of SPI/C/MDI, with PSPR_1_ and PSPR_2_ reduced to 0.039 m^2^/s and 0.011 m^2^/s, corresponding to decreases of 4.9% and 75.0%, respectively. SPI/C/MDI/IFR had a TSP of 2.44 m^2^, 28.7% lower than that of SPI/C/MDI, with PSPR_1_ and PSPR_2_ values of 0.043 m^2^/s and 0.023 m^2^/s; in this case, PSPR_1_ was 4.9% higher than that of SPI/C/MDI, whereas PSPR_2_ was 47.7% lower. Moreover, SPI/C/MDI/C@SiAPP18 reached PSPR_1_ at 70 s, 10–20 s later than the other three samples, and reached PSPR_2_ at 1070 s, indicating that this flame-retardant system maintained effective smoke-suppression behavior throughout the middle-to-late stage of combustion.

To further demonstrate the performance of the proposed system, the flame-retardant and bonding performances of the SPI/C/MDI/C@SiAPP18-bonded plywood were compared with recently reported fireproof systems based on soy protein adhesives or flame-retardant plywood, as summarized in Table 4. Wu et al. [6] prepared a flame-retardant decorated plywood using an intumescent adhesive composed of APP, pentaerythritol, and melamine–urea–formaldehyde resin, which reduced the average heat release rate and the total heat release within 300 s by 89.3% and 88.6%, respectively; however, that system relied on a formaldehyde-based resin. Xu et al. [16] developed a soy protein adhesive reinforced with a graphene-based organic–inorganic hybrid and phytic acid, achieving a limiting oxygen index (LOI) of 35.5% for the cured adhesive. Zhang et al. [29] reported a soy protein adhesive crosslinked with a POSS–urushiol core–shell hybrid and sodium borate, which reduced the PHRR of the cured adhesive by 25.4% and provided dry and wet shear strengths of 2.46 and 0.74 MPa, respectively. Notably, the flame retardancy in those studies was evaluated at the adhesive level rather than on the bonded plywood. Yang et al. [15] achieved a plywood LOI of 38.5% by combining a dialdehyde cellulose-based adhesive with a phytic acid/SiO_2_ veneer coating, but this required an additional surface-treatment step applied to the veneers. In comparison, the P–N–Si intumescent system proposed in this work is incorporated entirely within the formaldehyde-free adhesive layer without any veneer pretreatment, and the resulting plywood itself exhibited reductions of 23.5%, 20.7%, and 43.9% in PHRR, THR, and TSP, respectively, under cone calorimeter testing, while maintaining dry and wet shear strengths of 1.15 and 0.81 MPa that satisfy the Class II requirement of GB/T 9846-2015. This comparison demonstrates that the proposed system offers a well-balanced combination of bonding performance, flame retardancy, and smoke suppression at the plywood level.

### 3.4. Smoke-Gas Analysis of the Plywood

As shown in Figure 5 and Table 5, SPI/C/MDI exhibited peak CO production rates (P-COP_1_ and P-COP_2_) of 0.00373 g/s and 0.00554 g/s, respectively, with a total organic carbon release (TOC) of 48.34 g. SPI/C/MDI/C@SiAPP18 showed P-COP_1_ and P-COP_2_ values of 0.00287 g/s and 0.00217 g/s, representing reductions of 23% and 60.8% relative to SPI/C/MDI, and its TOC was 38.29 g, 20.8% lower than that of SPI/C/MDI. SPI/C/MDI/C@SiAPP15 exhibited P-COP_1_, P-COP_2_, and TOC values of 0.00279 g/s, 0.00422 g/s, and 40.42 g, respectively, of which P-COP_2_ and TOC were slightly higher than those of SPI/C/MDI/C@SiAPP18, whereas all three values remained lower than those of SPI/C/MDI. SPI/C/MDI/IFR showed P-COP_1_ and P-COP_2_ values of 0.00558 g/s and 0.00388 g/s, respectively, along with a relatively high TOC value. Except for SPI/C/MDI/C@SiAPP18, the other three samples reached P-COP1 at about 60 s, whereas SPI/C/MDI/C@SiAPP18 reached P-COP_1_ at 70 s and P-COP_2_ at 1200 s, later than all the other samples.

For CO_2_ release, SPI/C/MDI/C@SiAPP18 exhibited peak CO_2_ production rates (P-CO_2_P_1_ and P-CO_2_P_2_) of 0.099 g/s and 0.147 g/s, corresponding to reductions of 51% and 22% relative to SPI/C/MDI. SPI/C/MDI/C@SiAPP15 showed a P-CO_2_P_2_ of 0.178 g/s, slightly higher than that of SPI/C/MDI/C@SiAPP18, whereas SPI/C/MDI/IFR reached 0.165 g/s, falling between the values of SPI/C/MDI/C@SiAPP18 and SPI/C/MDI/C@SiAPP15. Similar to the CO release behavior, SPI/C/MDI/C@SiAPP15 and SPI/C/MDI/IFR reached P-CO_2_P_1_ at about 60 s, whereas SPI/C/MDI/C@SiAPP18 reached this peak at 70 s and reached its second CO_2_ peak at 1040 s, again later than the other three samples. These results indicate that, among the tested formulations, SPI/C/MDI/C@SiAPP18 achieved the greatest simultaneous reduction in CO and CO_2_ release rates, followed by SPI/C/MDI/C@SiAPP15, which still performed better than the conventional flame-retardant system SPI/C/MDI/IFR.

### 3.5. TGA-FTIR Analysis of the Flame-Retardant Adhesive

Figure 6 shows the 3D TGA-FTIR curves of the thermo-oxidative decomposition products of SPI/C/MDI and SPI/C/MDI/C@SiAPP18, along with the corresponding FTIR spectra recorded at different temperatures. The main thermo-oxidative decomposition products of SPI/C/MDI were H_2_O (3737 cm^−1^), CO_2_ (2364 cm^−1^ and 2329 cm^−1^), C=O-containing compounds (1741 cm^−1^), and C-H-containing compounds (2940 cm^−1^ and 1512 cm^−1^). Because SPI/C/MDI contained collagen, ammonia (962 cm^−1^ and 928 cm^−1^) was also detected among the thermo-oxidative decomposition products. In contrast, the thermo-oxidative decomposition products of SPI/C/MDI/C@SiAPP18 differed markedly: P=O (1270 cm^−1^) and P-O (1079 cm^−1^) bond signals appeared after 330 °C, and the intensity of the ammonia bands increased, which may be attributed to phosphate cleavage and the accompanying release of ammonia. In addition, according to the previous literature reports [29,30], such phosphorus-containing decomposition may further exert potential gas-phase flame-retardant effects: the released phosphorus-containing radicals (e.g., PO· and HPO·) may quench active H·, O·, and HO· radicals and thereby terminate the combustion chain reactions, while also facilitating the conversion of CO to CO_2_ and reducing the generation of combustible gases. Meanwhile, non-combustible gases such as water vapor, CO_2_, and ammonia exert a dilution effect during the critical stage of char formation, assisting the adhesive in forming a dense char layer and thereby producing a condensed-phase/gas-phase synergistic flame-retardant effect.

### 3.6. Flame-Retardant Mechanism Analysis

As shown in Figure 7, only a small amount of severely fragmented char residue remained on SPI/C/MDI after combustion, whereas the char residue of SPI/C/MDI/C@SiAPP18 formed a dense and continuous structure with film-like or granular deposits on the surface. EDS analysis showed that the char residue of SPI/C/MDI/C@SiAPP18 was mainly composed of C, O, P, and Si, accounting for 62.1%, 33.0%, 4.7%, and 0.2%, respectively, indicating the formation of inorganic products such as phosphates and silicon oxides during combustion. These inorganic species acted synergistically with the pyrolysis products of cellulose and lignin to improve char compactness and oxidation resistance, thereby forming an effective thermal barrier.

Figure 8a presents the FTIR spectra of the char residues of SPI/C/MDI and SPI/C/MDI/C@SiAPP18. SPI/C/MDI showed pronounced C=O (1577 cm^−1^), C-H (1400 cm^−1^), and C-O-C (1104 cm^−1^) vibration bands, whereas the char residue of SPI/C/MDI/C@SiAPP18 exhibited a weaker C-H band and a stronger C=O band. This result suggests that the flame-retardant system promoted dehydrogenation and oxidative crosslinking of the wood components, facilitating the conversion of aliphatic structures into more stable aromatic structures and thereby improving char compactness and thermal stability. The P-O-C band (1078 cm^−1^) detected in the char residue of SPI/C/MDI/C@SiAPP18 can be attributed to an in situ phosphorylation reaction between the wood matrix and phosphate groups released during thermal cleavage of the flame retardant. The appearance of the P-N band (950 cm^−1^) further indicates crosslinking reactions within the char layer, in which the P-N crosslinked network enhances the structural strength of the residue. Si-O-Si (780 cm^−1^) and Si-C (860 cm^−1^) bands were also observed in the char residue of SPI/C/MDI/C@SiAPP18, indicating that the silane-modified component condensed at high temperature to form a crosslinked siloxane network and anchored inorganic silicon within the char layer through Si-C bonds. This structure improves the compactness, thermal stability, and oxidation resistance of the char layer, thereby strengthening its physical barrier effect.

Figure 8b,c show the Raman spectra of the char residues of SPI/C/MDI and SPI/C/MDI/C@SiAPP18. The I_D_/I_G_ ratio decreased from 3.77 for SPI/C/MDI to 1.81 for SPI/C/MDI/C@SiAPP18, indicating that treatment with the nitrogen-phosphorus-silicon compounds transformed the char generated during combustion from a loose and disordered structure into a more ordered, graphite-like structure. This more ordered and graphitic char provides stronger thermal-barrier properties, effectively shielding the condensed phase from external heat penetration and blocking the release pathways of flammable volatiles from the interior.

Figure 8d–g show the XPS spectra of the char residues of SPI/C/MDI and SPI/C/MDI/C@SiAPP18. The char residue of SPI/C/MDI/C@SiAPP18 preserved the N, P, and Si flame-retardant elements, indicating that part of the flame-retardant components was retained in the condensed phase during combustion. In the N 1s spectrum, the peaks at 398.8, 400.5, and 404.4 eV were assigned to C=N–C, C–NH, and oxidized nitrogen species such as N–O, respectively. The C=N–C and C–NH structures suggest that the nitrogen-containing components underwent condensation and aromatization during combustion, contributing to the formation of nitrogen-containing heterocyclic char structures. In the P2p spectrum, the peaks at approximately 134.0 and 134.7 eV were assigned to P–N and P–O–C, respectively, indicating that phosphorus-containing species participated in crosslinking and esterification reactions during char formation. In the Si2p spectrum, the peaks at 101.9 and 103.6 eV were assigned to Si–P/Si–N and Si–O, respectively, suggesting that silicon was retained in the char layer in the form of silicon–oxygen structures and phosphorus–silicon/nitrogen–silicon hybrid structures. Overall, these species promoted the formation of a multi-element hybrid char layer containing nitrogen-containing aromatic structures, phosphate species, and silicon–oxygen networks, thereby improving the continuity and stability of the protective char layer.

The above experimental results, including the SEM-EDS, FTIR, Raman, and XPS analyses of the char residues together with the cone calorimeter data, consistently indicate that condensed-phase char formation and structural strengthening constitute the principal flame-retardant mechanism of the C@Si@APP system, which can be summarized as follows (Figure 9). In the condensed phase, catalytic char formation and structural strengthening were promoted by the system. Cellulose, lignin, and hemicellulose in wood mainly supplied C-C bonds and -OH groups. Reactive polyphosphoric species produced from C@Si@APP promoted dehydration and charring of soy protein and lignin, while an in situ phosphorylation reaction formed P-O-C linkages. Together with the P-N covalent network generated from the nitrogen source, these reactions further improved the stability of the char layer. At elevated temperature, the silicon component migrated to the surface and condensed into a crosslinked siloxane network (Si-O-Si). Inorganic silicon was incorporated into the char framework through Si-C bonds, which further enhanced the thermal stability and oxidation resistance of the char. Finally, under the combined catalytic effect of phosphorus, nitrogen, and silicon, the initially disordered thermal-decomposition products of the biomass were transformed into a more ordered, graphite-like structure, resulting in the formation of a dense, continuous, high-temperature-resistant, multiscale composite protective char layer that effectively reduced the transfer of heat and combustible volatiles.

In addition to the condensed-phase mechanism demonstrated above, a gas-phase contribution can be reasonably postulated, although it was not directly verified in this study. The TGA-FTIR results (Section 3.5) indicated the release of non-combustible gases such as NH_3_, H_2_O, and CO_2_ from the decomposition of C@Si@APP and the protein components; these gases may act as diluting agents during the initial stage of combustion, potentially reducing the concentrations of oxygen and combustible species near the surface. Meanwhile, as proposed in previous studies [35,36], phosphorus-containing radicals such as PO· and HPO· may be released from the phosphorus component and could scavenge reactive HO· and O· radicals in the gas phase, thereby potentially interrupting the combustion chain reaction and limiting sustained combustion. Therefore, the gas-phase effect is regarded here as a plausible complementary mechanism inferred from the TGA-FTIR results and the existing literature, rather than a mechanism directly demonstrated by the present experimental data.

## 4. Conclusions

In this study, a self-synthesized C@Si@APP flame retardant was blended with soy protein, industrial collagen, and MDI to prepare a high-performance, formaldehyde-free soy-based flame-retardant adhesive, used as the bonding layer for eucalyptus veneers to produce seven-layer flame-retardant plywood. Among the tested formulations, the plywood bonded with SPI/C/MDI/C@SiAPP18 showed the most effective flame-retardant and smoke-suppression performance. In addition, the plywood bonded with SPI/C/MDI/C@SiAPP18 exhibited dry and wet shear strengths of 1.15 MPa and 0.81 MPa, respectively, satisfying the bonding-strength requirements for Class II interior-use plywood (≥0.7 MPa) specified in GB/T 9846-2015.

Compared with the control plywood (SPI/C/MDI), SPI/C/MDI/C@SiAPP18 exhibited a PHRR_1_ of 222.6 kW/m^2^ and a THR_1170s_ of 50.45 MJ/m^2^, corresponding to reductions of 23.5% and 20.7%, respectively, and it also performed better than the conventional flame-retardant system SPI/C/MDI/IFR. For smoke and gas suppression, its TSP was 43.9% lower than that of SPI/C/MDI, the release rates of CO and CO_2_ were both reduced, and TOC decreased from 48.34 g to 38.29 g.

These improvements are mainly attributed to a condensed-phase char-forming mechanism supported by the experimental evidence. The characteristic P-O-C and P-N bands detected in the char residue suggest that phosphate groups may have catalyzed an in situ phosphorylation reaction within the wood components and, together with the crosslinked siloxane network formed by the silicon component, promoted the conversion of aliphatic wood structures into a more ordered, graphite-like char layer, thereby effectively blocking the transfer of heat and oxygen. In addition, a complementary gas-phase contribution is plausible: the non-combustible gases (NH_3_, H_2_O, and CO_2_) detected by TGA-FTIR may have diluted the oxygen and combustible-gas concentration, and phosphorus-containing radicals (PO·, HPO·) were considered capable of scavenging reactive radicals in the gas phase. It should be noted that the gas-phase pathways were inferred from the TGA-FTIR results and the previous literature rather than directly demonstrated, and further work is needed to quantify their contribution.

## Figures and Tables

**Figure 1:**
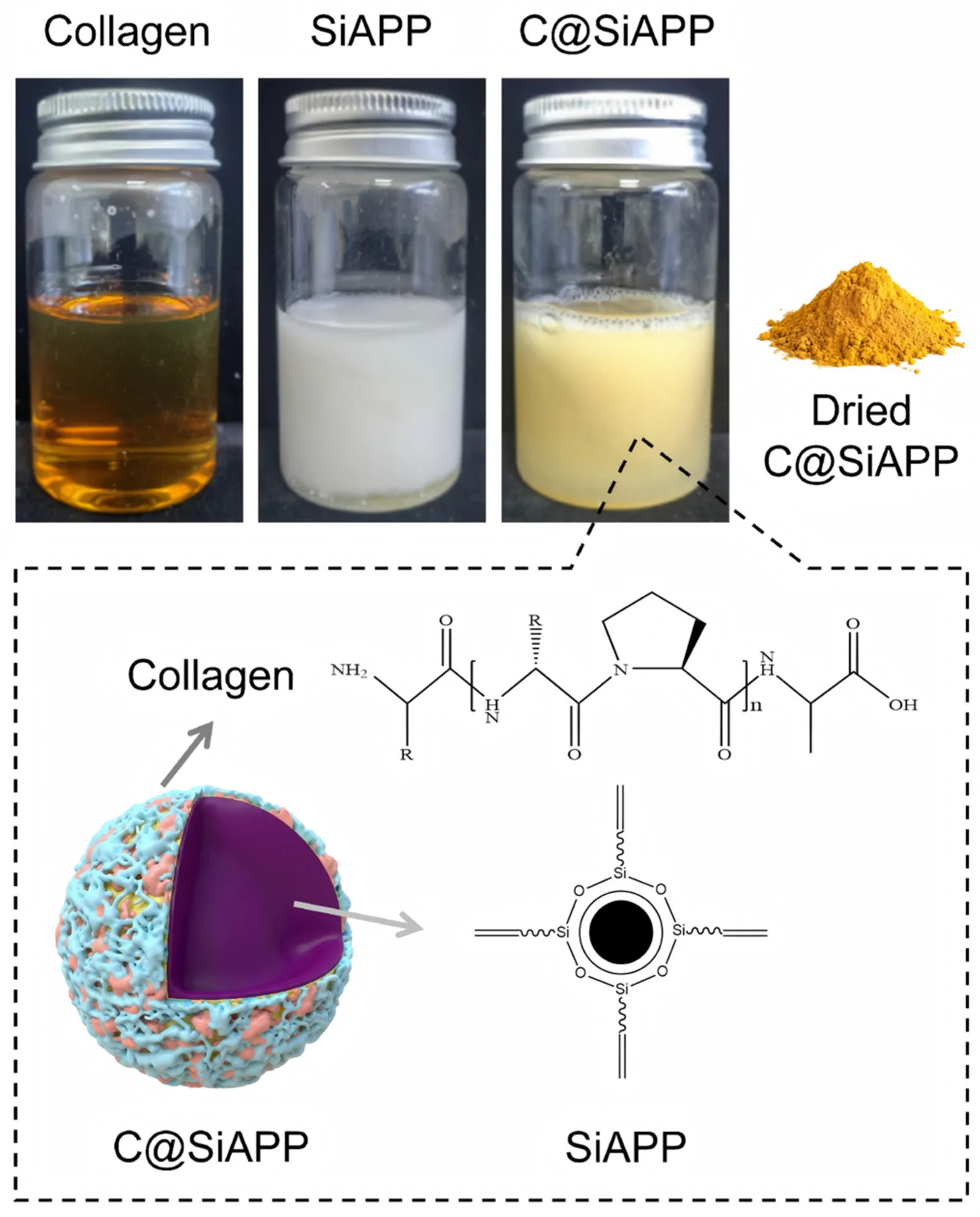
Preparation illustration of C@SiAPP.

**Figure 2:**
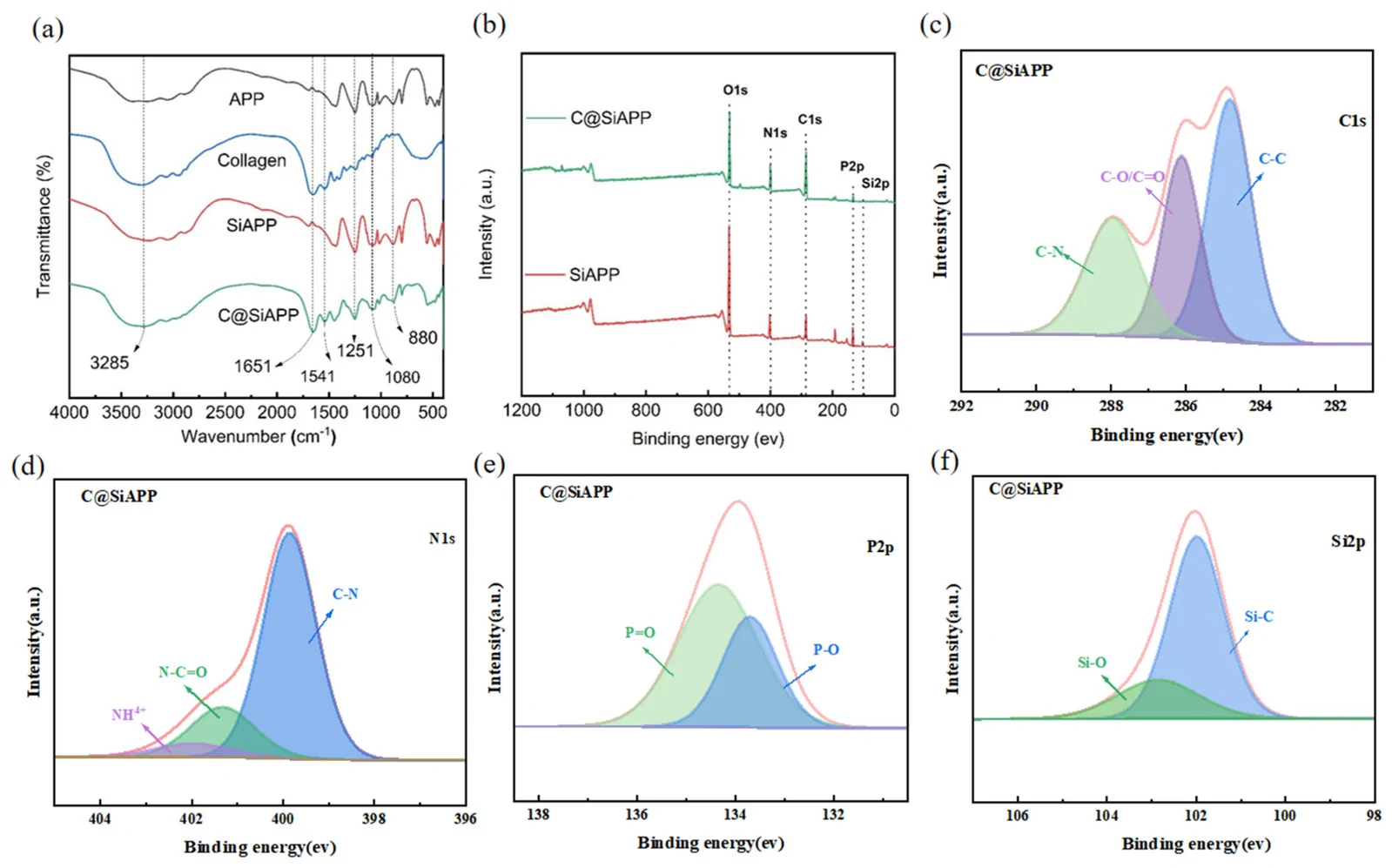
(**a**) FTIR spectra of APP, Si@APP, collagen, and C@Si@APP. (**b**) XPS survey spectra of Si@APP and C@Si@APP. (**c**) C1s, (**d**) N1s, (**e**) P2p, and (**f**) Si2p spectra of C@Si@APP.

**Figure 3:**
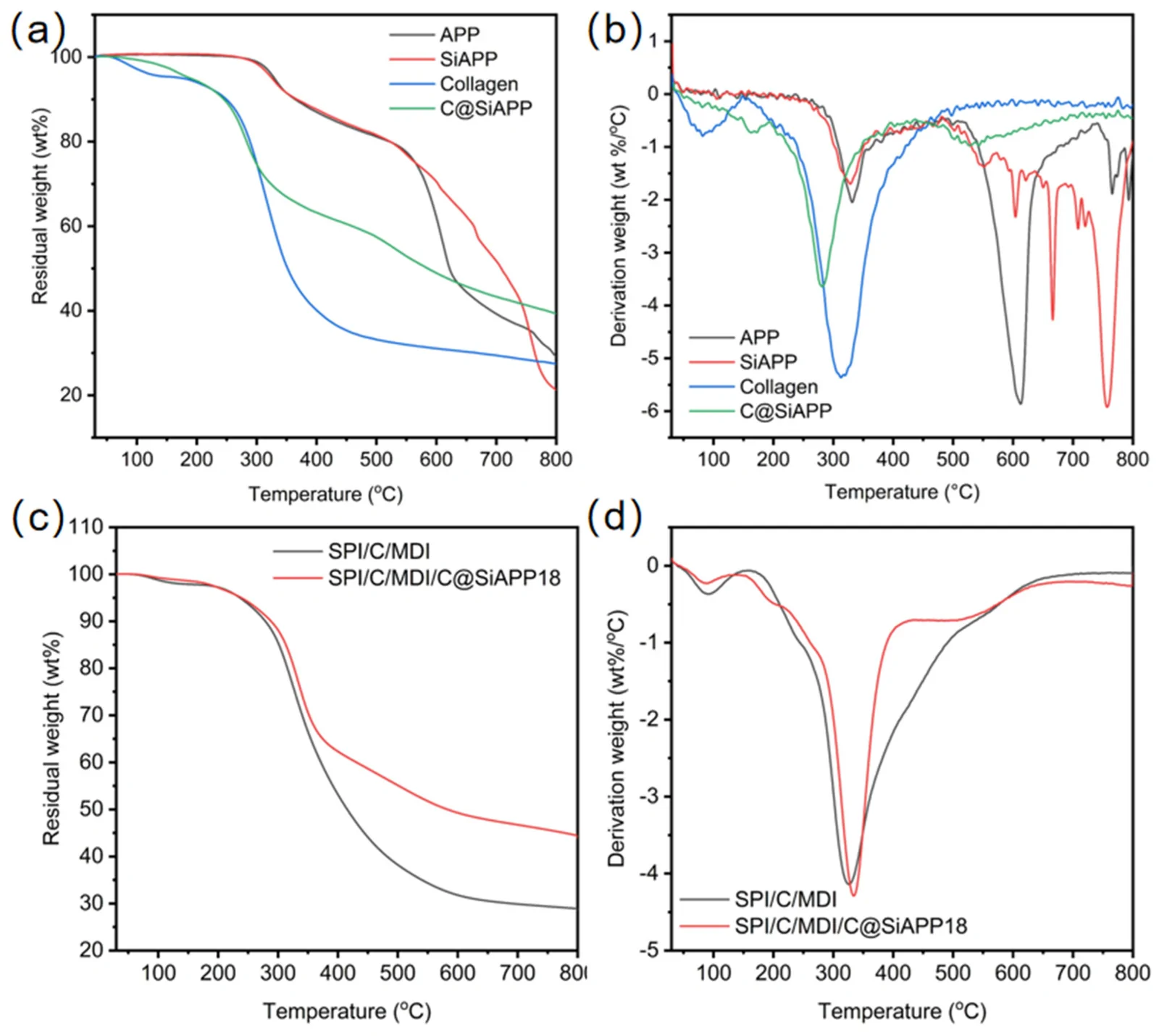
(**a**) TG and (**b**) DTG curves of APP, Si@APP, collagen, and C@Si@APP. (**c**) TG and (**d**) DTG curves of SPI/C/MDI and SPI/C/MDI/C@SiAPP18.

**Figure 4:**
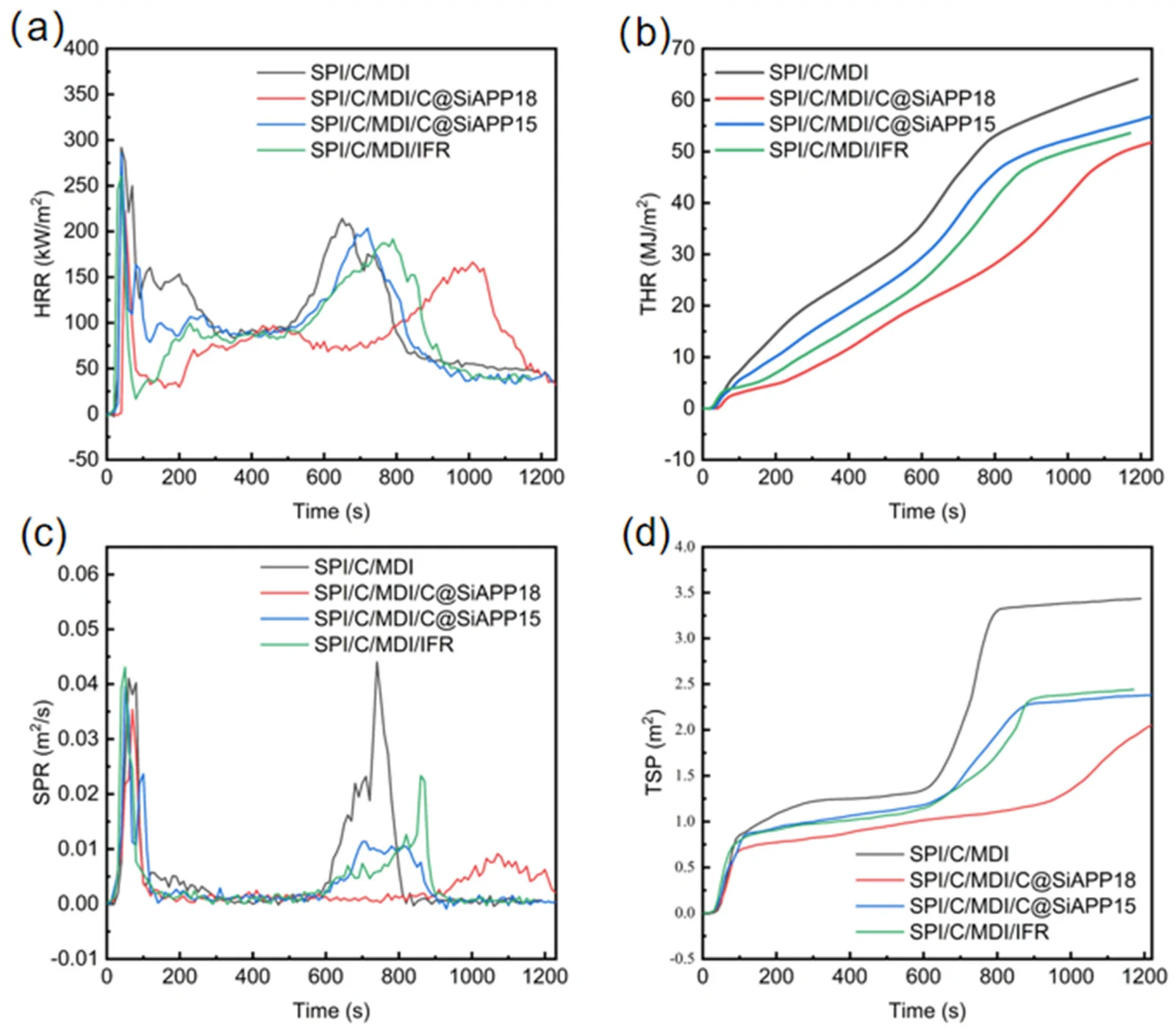
(**a**) THR, (**b**) HRR, (**c**) SPR, and (**d**) TSP curves of SPI/C/MDI, SPI/C/MDI/C@SiAPP18, SPI/C/MDI/C@SiAPP15, and SPI/C/MDI/IFR.

**Figure 5:**
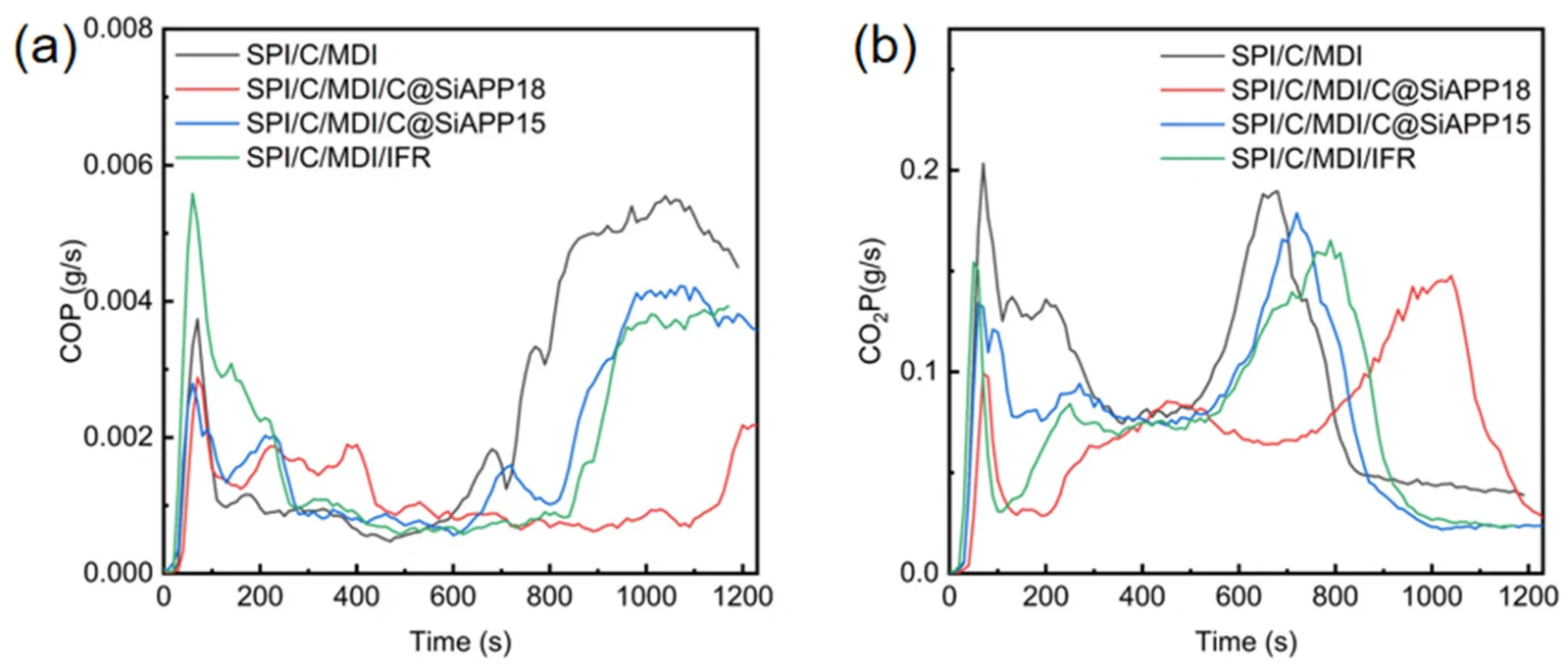
(**a**)COP, (**b**) CO_2_P curves of SPI/C/MDI, SPI/C/MDI/C@SiAPP18, SPI/C/MDI/C@SiAPP15, and SPI/C/MDI/IFR.

**Figure 6:**
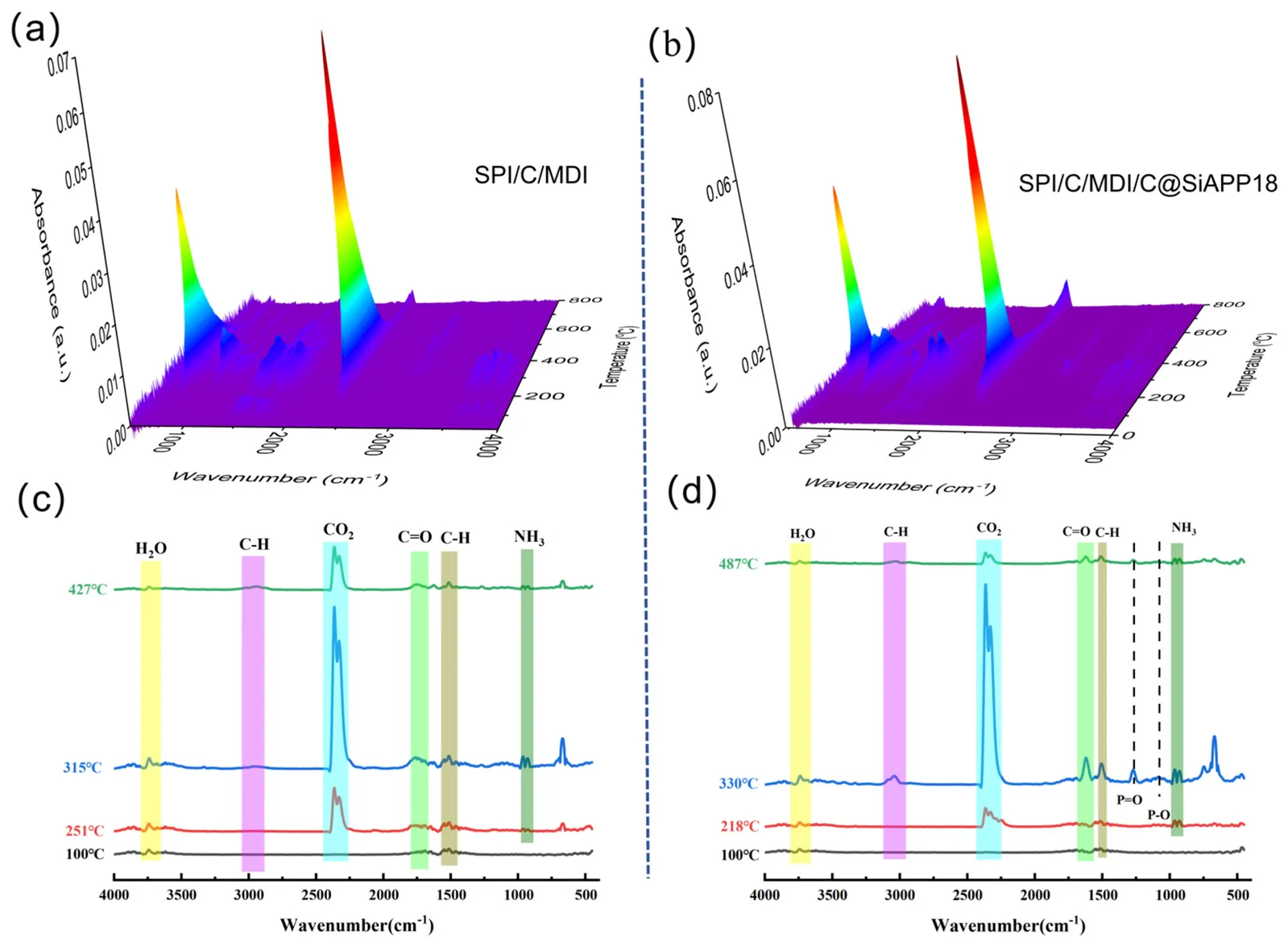
3D TGA-FTIR curves and corresponding FTIR spectra at different temperatures for the thermo-oxidative decomposition products of (**a**,**c**) SPI/C/MDI and (**b**,**d**) SPI/C/MDI/C@SiAPP18.

**Figure 7:**
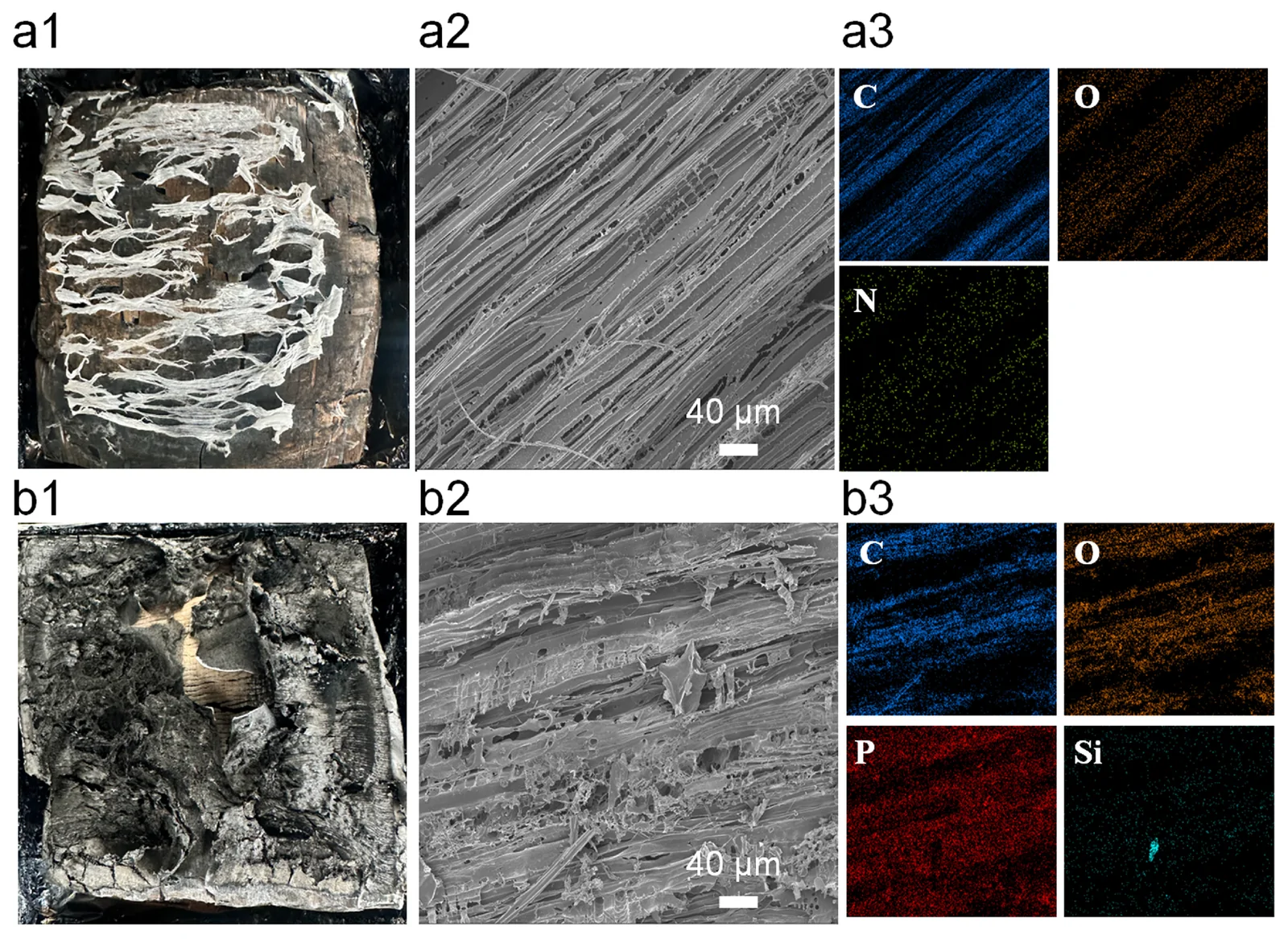
SEM images and EDS elemental mapping of the char residues of (**a**) SPI/C/MDI (**a1**–**a3**) and (**b**) SPI/C/MDI/C@SiAPP18 (**b1**–**b3**).

**Figure 8:**
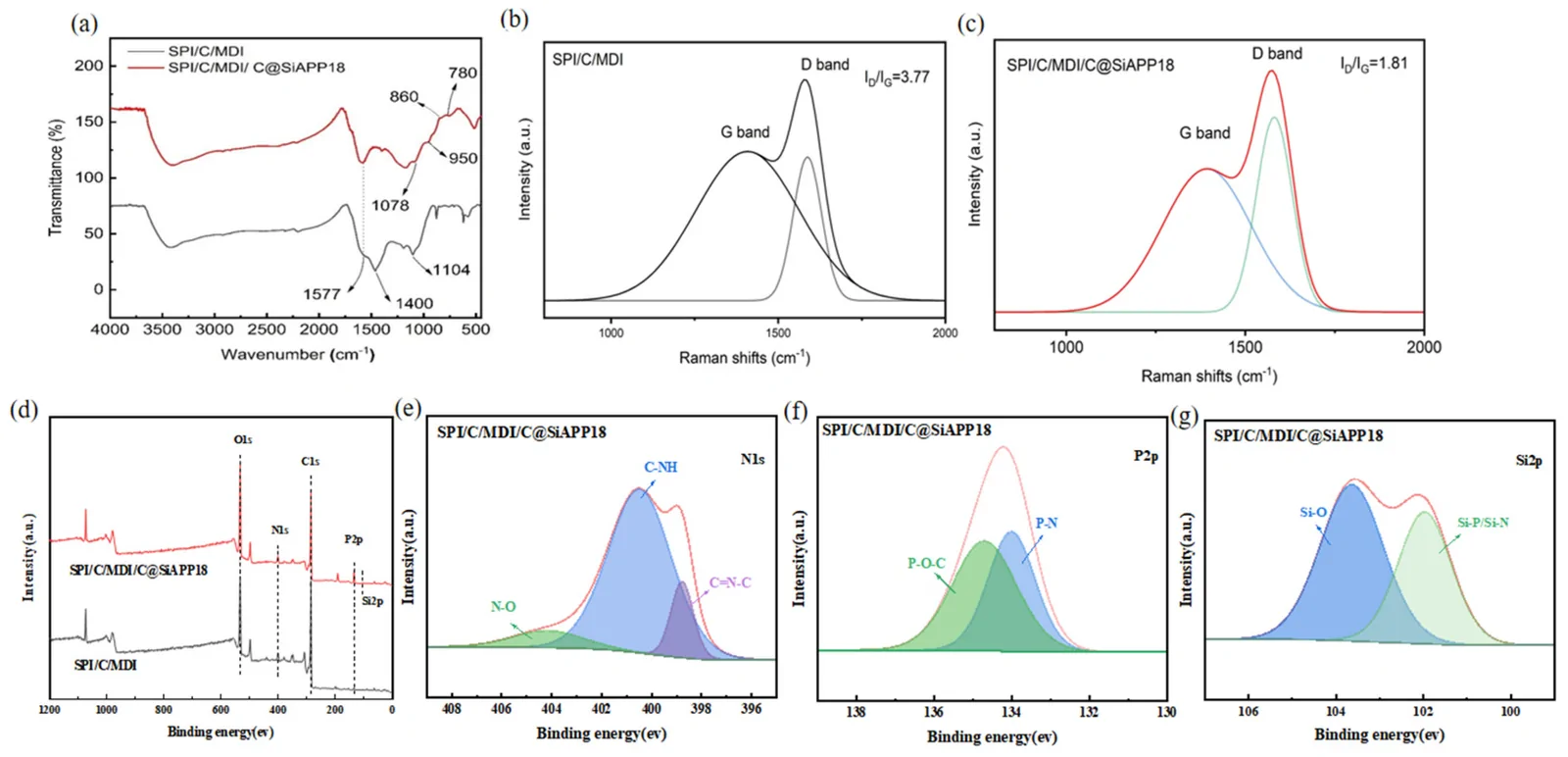
(**a**) FTIR spectra, (**b**,**c**) Raman spectra, and (**d**–**g**) XPS spectra of the char residues of SPI/C/MDI and SPI/C/MDI/C@SiAPP18.

**Figure 9:**
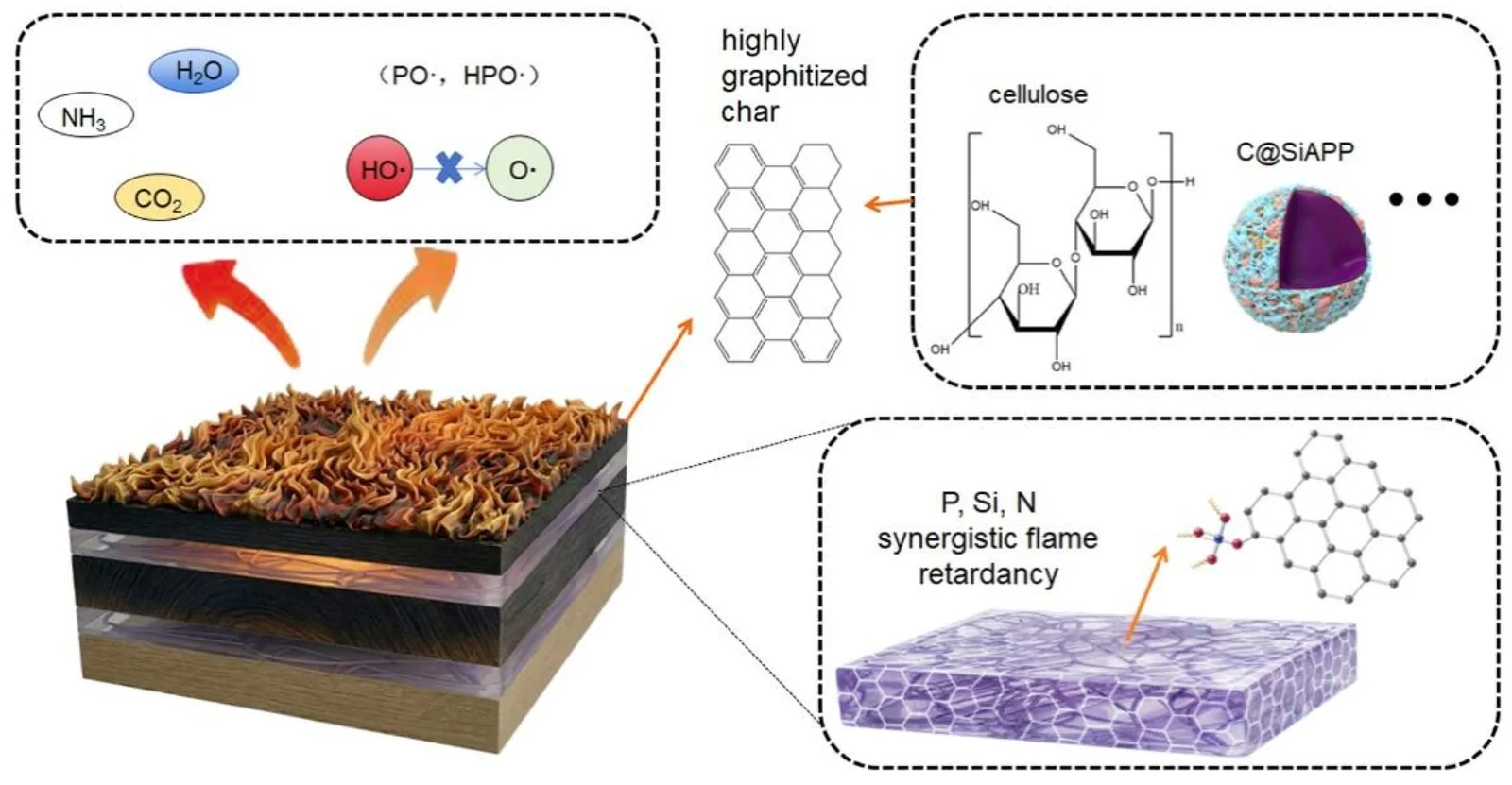
Flame retardant mechanism diagram of SPI/C/MDI/C@SiAPP18.

**Table 1:** Formulations of the soy protein-based flame-retardant composite adhesives (parts by weight). The numbers 18 and 15 in the sample codes denote the parts by weight of C@Si@APP in the corresponding formulations (per 70 parts of the SPI/Col/MDI base adhesive), and the plywood specimens are denoted by the codes of the adhesives used for their preparation.

Sample Code	Composition Content						
	SPI	Col	MDI	IFR	C@Si@APP	Adenine	Lignin
SPI/C/MDI	64	16	20	/	/	/	/
SPI/C/MDI/C@SiAPP18	44.8	11.2	14	/	18	6	6
SPI/C/MDI/C@SiAPP15	44.8	11.2	14	/	15	10	5
SPI/C/MDI/IFR	44.8	11.2	14	30	/	/	/

Note: IFR refers to an intumescent flame retardant composed of ammonium polyphosphate (APP), melamine (MEL), and pentaerythritol (PER).

**Table 2:** Thermal decomposition parameters of the flame retardants and flame-retardant adhesives.

Sample	T_5%_ (°C)	T_max1_ (°C)	PMLR (%/°C)	T_max2_ (°C)	PMLR (%/°C)	W_exp-800 °C_ (%)
APP	329	322	2.03	613	5.84	29.1
Si@APP	325	328	1.69	757	5.92	21.2
Collagen	175	80	0.76	315	5.33	24.7
C@Si@APP	188	280	3.63	542	0.97	39.2
SPI/C/MDI/C@SiAPP18	238	88	0.22	335	4.20	44.2
SPI/C/MDI	236	92	0.36	326	4.10	28.8

**Table 3:** Cone calorimeter data for SPI/C/MDI, SPI/C/MDI/C@SiAPP18, SPI/C/MDI/C@SiAPP15, and SPI/C/MDI/IFR.

Sample	PHRR_1_ (kW/m^2^)	Time to PHRR_1_ (s)	PHRR_2_ (kW/m^2^)	Time to PHRR_2_ (s)	THR_1170s_ (MJ/m^2^)	PSPR_1_ (m^2^/s)	Time to PSPR_1_ (s)	PSPR_2_ (m^2^/s)	Time to PSPR_2_ (s)	TSP_1170s_ (m^2^)
SPI/C/MDI/C@SiAPP18	222.6	50	116	1010	50.45	0.035	70	0.009	1070	1.92
SPI/C/MDI/C@SiAPP15	285	40	203	720	55.61	0.039	50	0.011	710	2.37
SPI/C/MDI/IFR	260	40	191	790	53.58	0.043	50	0.023	860	2.44
SPI/C/MDI	291	40	214	650	63.59	0.041	60	0.044	740	3.42

**Table 4:** Comparison of the flame-retardant and bonding performances of the system proposed in this work with recently reported fireproof systems based on soy protein adhesives or flame-retardant plywood.

System	Flame-Retardant Strategy	Evaluation Level	Flame-Retardant Performance	Dry/Wet Shear Strength (MPa)	Ref.
MUF/APP/PER intumescent adhesive (flame-retardant decorated plywood)	P–N intumescent adhesive (APP/PER) in formaldehyde-based MUF resin	Plywood (cone calorimeter, 50 kW/m^2^)	AHRR and THR within 300 s reduced by 89.3% and 88.6% vs. untreated plywood; B1(B) class (GB/T 8624) [34]	–/>0.97	[6]
SPI/PA/G-co-Q soy protein adhesive	Graphene-based organic–inorganic hybrid with phytic acid	Cured adhesive (LOI)	LOI of 35.5% (49.1% higher than the SPI adhesive)	+76.6%/+78.7% vs. SPI	[16]
SPI/POSS-U/SB soy protein adhesive	POSS–urushiol core–shell hybrid with borate crosslinking	Cured adhesive (cone calorimeter)	PHRR reduced by 25.4% (from 283.1 to 211.3 kW/m^2^)	2.46/0.74	[29]
DPMP adhesive with PA/SiO_2_ veneer coating	Phytic acid/SiO_2_ coating on veneers combined with a bio-based adhesive	Plywood (LOI)	LOI of 38.5%	1.6/–	[15]
SPI/C/MDI/C@SiAPP18 plywood (this work)	P–N–Si intumescent flame retardant within a formaldehyde-free adhesive layer; no veneer pretreatment	Plywood (cone calorimeter, 50 kW/m^2^)	PHRR, THR, and TSP reduced by 23.5%, 20.7%, and 43.9% vs. SPI/C/MDI	1.15/0.81	This work

**Table 5:** CO and CO_2_ release data for SPI/C/MDI, SPI/C/MDI/C@SiAPP18, SPI/C/MDI/C@SiAPP15, and SPI/C/MDI/IFR.

Sample	P-COP_1_ (g/s)	Time to P-COP_1_ (s)	P-COP_2_ (g/s)	Time to P-COP_2_ (s)	TOC_1170s_ (g)	P-CO_2_P_1_ (g/s)	Time to P-CO_2_P_1_ (s)	P-CO_2_P_2_ (g/s)	Time to P-CO_2_P_2_ (s)
SPI/C/MDI/C@SiAPP18	0.00287	70	0.00217	1200	38.29	0.099	70	0.147	1040
SPI/C/MDI/C@SiAPP15	0.00279	60	0.00422	1070	40.42	0.134	60	0.178	720
SPI/C/MDI/IFR	0.00558	60	0.00388	1152	41.90	0.154	50	0.165	790
SPI/C/MDI	0.00373	60	0.00554	1040	48.34	0.203	70	0.189	680

## Data Availability

The original contributions presented in this study are included in the article. Further inquiries can be directed to the corresponding authors.
